# Metropolitan Hospital Sunday Fund

**Published:** 1895-11-02

**Authors:** 


### METROPOLITAN HOSPITAL SUNDAY FUND.

We have great pleasure in reproducing the following article from last week's *Lancet*. We joyfully welcome our contemporary's attitude in the matter, and feel confident that the cordial feelings expressed, which we warmly reciprocate, and the recognition of the fact that, after all, the work is everything and the individual workers practically nothing, is the only true principle which should animate reasonable men in public work. Unfortunately that is sometimes forgotten, or not understood; but the prospects of the Metropolitan Hospital Sunday Fund, which the hospitals owe mainly to the *Lancet*, must be brighter and better henceforward than ever before :—

The subscriptions to the Metropolian Hospital Sunday Fund have this year attained to a sum far in advance of any sum ever previously reached, and the result produced upon us is the same as that which the sight of the workhouse gruel produced upon Oliver Twist—we can only say (with due gratitude for what has been received), "Please, we want some more." And the combination of greed and gratitude is easily explained. For ten years the total sum received, while varying in its units, tens, and hundreds, had remained fairly constant in its thousands, and we had, in common with the officials of the Fund, begun to believe that the high-water mark was fixed at, say, £42,000. In good times the figure would rise, as in bad times it would fall, but this sum seemed to represent the amount which the public were prepared to give, though we have never allowed that it was quite the sum that should be expected, having regard to the size of the community and the undeniable excellence of the Fund. We had almost begun to believe that all efforts were required to bring the Fund to the point which it usually reached, and that much beyond that point it was useless to expect contributions, though a drop to much below that point might occur if the exertions of the supporters of the Fund were ever so little relaxed. The Fund, however, this year has reached the noble sum of £60,000—a leap by £15,000 in advance of the previous maximum sum. This striking proof that there is nothing constant about the figure to be expected, nothing stereotyped about the generosity of the public towards the support of our metropolitan hospitals, at once emboldens us to ask: If £60,000 this year, why not £70,000 another year? And it leads us also to look into the origin of the remarkable aggrandisement that has taken place in 1895, that we may learn its *rationale*, and apply, or assist in applying, similar methods on future occasions towards furthering the interests of a Fund whose object we have so much at heart, and with whose foundation and maintenance we can claim to have been so closely connected. The upward bound has certainly been due to the action of our contemporary The Hospital, a journal devoted to the interests of our hospitals and asylums, which published this year a special appeal that resulted in securing subscriptions to the Fund amounting to no less than £18,000, and we heartily congratulate our contemporary, no less than the Fund, upon this splendid outcome of well-directed journalism. The sum so raised represents the increase of the year, for the collections in the churches and from the public at large by offertory and donation remain the same as before—hardly, indeed, reaching the figure we have set down as the decennial average. But the large sums received through Messrs. Burdett and Harris, and Messrs. Pym and Vaughan from the Stock Exchange, and the princely generosity of Mr. B. Barnato, Mr. D. Marks, "and other City friends"—as they modestly style themselves in their letter to Mr. Burdett—have raised the total of the Fund from its level, possibly becoming a dead level, to the splendid figure we have announced. And the source from which these huge donations were received suggests the method by which we must, one and all, attempt to keep the Fund in the position to which it has attained, and to avoid the retrogression that at the first glance may seem likely to occur. Mr. Burdett, the editor of The Hospital, is also Secretary to the Stock and Share Department of the Stock Exchange, and has been able to move that notoriously open-handed and open-hearted body of gentlemen to this extent, bringing to bear upon their sentiments and their purses the influence of personal and intimate knowledge. And this is the only way in which the Fund can be made to grow; we are not even certain that it is not the only way that it can be prevented from shrinking. Individual effort is wanted. Workers are wanted, zealots and proselytisers. And if by chance these can be found among the men who direct the paths of journals with power among different classes, sects, and *coteries* the success of the Fund, which all allow should be unquestioned, will be assured.

